# Transcriptome analyses reveal SR45 to be a neutral splicing regulator and a suppressor of innate immunity in *Arabidopsis thaliana*

**DOI:** 10.1186/s12864-017-4183-7

**Published:** 2017-10-11

**Authors:** Xiao-Ning Zhang, Yifei Shi, Jordan J. Powers, Nikhil B. Gowda, Chong Zhang, Heba M. M. Ibrahim, Hannah B. Ball, Samuel L. Chen, Hua Lu, Stephen M. Mount

**Affiliations:** 1 Biochemistry Program, Department of Biology, St. Bonaventure University, St. Bonaventure, NY 14778 USA; 2 CMNS-Institute for Advanced Computer Studies, University of Maryland, College Park, MD 20742 USA; 3 Department of Cell Biology and Molecular Genetics and Center for Bioinformatics and Computational Biology, University of Maryland, College Park, MD 20742 USA; 4 Biochemistry Program, St. Bonaventure University, St. Bonaventure, NY 14778 USA; 5 Department of Biology, St. Bonaventure University, St. Bonaventure, NY 14778 USA; 6 Department of Biological Sciences, University of Maryland Baltimore County, Baltimore, MD 21250 USA; 7 Genetics Department, Faculty of Agriculture, Cairo University, Cairo, Egypt; 8 Bioinformatics Program, St. Bonaventure University, St. Bonaventure, NY 14778 USA

**Keywords:** SR45, Differential gene expression, Regulation of alternative splicing, SR45-associated RNAs, Plant defense, Inflorescence

## Abstract

**Background:**

Regulation of pre-mRNA splicing diversifies protein products and affects many biological processes. *Arabidopsis thaliana* Serine/Arginine-rich 45 (SR45), regulates pre-mRNA splicing by interacting with other regulatory proteins and spliceosomal subunits. Although SR45 has orthologs in diverse eukaryotes, including human RNPS1, the *sr45–1* null mutant is viable. Narrow flower petals and reduced seed formation suggest that SR45 regulates genes involved in diverse processes, including reproduction. To understand how SR45 is involved in the regulation of reproductive processes, we studied mRNA from the wild-type and *sr45–1* inflorescences using RNA-seq, and identified SR45-bound RNAs by immunoprecipitation.

**Results:**

Using a variety of bioinformatics tools, we identified a total of 358 SR45 differentially regulated (SDR) genes, 542 SR45-dependent alternative splicing (SAS) events, and 1812 SR45-associated RNAs (SARs). There is little overlap between SDR genes and SAS genes, and neither set of genes is enriched for flower or seed development. However, transcripts from reproductive process genes are significantly overrepresented in SARs. In exploring the fate of SARs, we found that a total of 81 SARs are subject to alternative splicing, while 14 of them are known Nonsense-Mediated Decay (NMD) targets. Motifs related to GGNGG are enriched both in SARs and near different types of SAS events, suggesting that SR45 recognizes this motif directly. Genes involved in plant defense are significantly over-represented among genes whose expression is suppressed by SR45, and *sr45–1* plants do indeed show enhanced immunity.

**Conclusion:**

We find that SR45 is a suppressor of innate immunity. We find that a single motif (GGNGG) is highly enriched in both RNAs bound by SR45 and in sequences near SR45- dependent alternative splicing events in inflorescence tissue. We find that the alternative splicing events regulated by SR45 are enriched for this motif whether the effect of SR45 is activation or repression of the particular event. Thus, our data suggests that SR45 acts to control splice site choice in a way that defies simple categorization as an activator or repressor of splicing.

**Electronic supplementary material:**

The online version of this article (10.1186/s12864-017-4183-7) contains supplementary material, which is available to authorized users.

## Background

In eukaryotic cells, pre-mRNA splicing is an important posttranscriptional processing step in the production of mature mRNAs. Regulated alternative splicing is a ubiquitous mechanism that contributes to protein diversity and function in affected biological processes. During splicing, different spliceosome components are recruited in a stepwise fashion to achieve recognition of splice sites, activation, and catalysis. At the end, the introns are excised, the spliced product is released, and the spliceosome disassembles to complete one splicing event. This process involves many regulatory proteins, including SR protein splicing factors [1], and the Arabidopsis protein SR45 has been shown to complement human splicing extracts deficient in SR proteins [2]. The human ortholog of SR45 is RNPS1 [3], which was originally discovered as a splicing activator in human HeLa cells [4], and which interacts with spliceosome components [5]. In addition to its role in splicing, RNPS1 also functions in nonsense-mediated decay (NMD), a quality control step that is regulated by the exon junction complex (EJC). EJC is a conserved protein complex composed of four highly conserved core proteins and a suite of dynamic peripheral proteins, including RNPS1. In particular, RNPS1 couples the EJC to the UP-FRAMESHIFT (UPF) protein complex to trigger NMD [6]. These dual roles make RNPS1 a key bridge between splicing and the RNA quality control machinery, and it is likely that these functions of RNPS1 are conserved in SR45, its ortholog.

Use of alternative 3′ splice sites in the sixth intron of the *SR45* pre-mRNA results in two different mature transcripts, *SR45.1* and *SR45.2*. This leads to a seven amino acid difference between the two respective protein isoforms, SR45.1 and SR45.2. These two isoforms play distinct roles during plant growth and development [3]. A single phosphorylation event on threonine 218 (T^218^) within the distinguishing seven amino acids is crucial for the function of SR45.1 in flower petal development [7]. SR45 has two serine/arginine-serine-rich (S/RS) domains, which may be used for protein-protein interactions [8]. To-date, several SR45.1-interacting proteins, including spliceosomal proteins and splicing factors such as SR proteins, have been identified using in vivo and/or in vitro approaches [7, 9–11]. It is likely that SR45 interacts with these proteins through RS domains and functions to recruit snRNPs as in animals during the initiation and later steps of spliceosome assembly on target transcripts. In addition, SR45 co-localizes with EJC core proteins in the nucleolus [12], especially during hypoxia [13]. All of this evidence reveals a complex protein-protein interaction network that involves SR45 and can potentially increase transcript complexity dramatically.

A null *Arabidopsis* mutant, *sr45–1*, is viable as a homozygote, but exhibits various growth and developmental defects, including delayed root growth, narrower leaves and flower petals, late flowering, and unusual numbers of floral organs [2]. Interestingly, the late flowering phenotype of the *sr45–1* mutant is not only due to a lack of expression of *Flower Locus C* (*FLC*), but is also associated with insufficient DNA methylation in the RNA-dependent DNA methylation (RdDM) pathway [14]. This suggests that SR45 may regulate RdDM components for proper gene silencing during reproduction. In addition, the *sr45–1* mutant is hypersensitive to abiotic conditions, such as glucose and ABA [15, 16], suggesting that SR45 regulates not only genes involved in growth and development, but also genes that mediate responses to environmental changes. However, effects of SR45 on biotic stresses have not been reported.

The precise role of SR45 in the regulation of RNA processing remains unknown. Indeed, the pleiotropic phenotypes revealed by the *sr45–1* mutant indicate significant functional diversity among targets of regulation by SR45. In a recent study of early seedlings, Xing et al. [16] found that SR45 can associate with and regulate transcripts from genes involved in ABA signaling. This observation provides a mechanistic explanation for the ABA hypersensitivity of the *sr45–1* mutant [15].

To investigate whether and how SR45 regulates flower development to promote reproductive success, we subjected inflorescence tissue from two independent SR45.1-GFP transgenic lines to RNA-immunoprecipitation followed by high throughput sequencing (RIP-seq). We also performed RNA-seq analysis to compare the transcriptome of inflorescence tissue from wild type and *sr45–1* plants*.* Indeed, we find significant enrichment of reproduction-related genes among SARs. Other SARs have diverse molecular functions. This is consistent with a general role for SR45 in the processing of mRNA precursors. In addition, we find sequence motifs related to GGNGG to be enriched in both specific RIP-seq reads associated with SR45 and regions flanking sites of SR45-dependent alternative splicing. Furthermore, defense response genes were highly enriched in the SR45-suppressed genes, and this unexpected role of SR45 in defense response was further confirmed by plant defense assays, revealing an unexpected role for SR45 in suppressing plant innate immunity.

## Results

### SR45-regulated differential gene expression in inflorescence

**Fig. 1:**
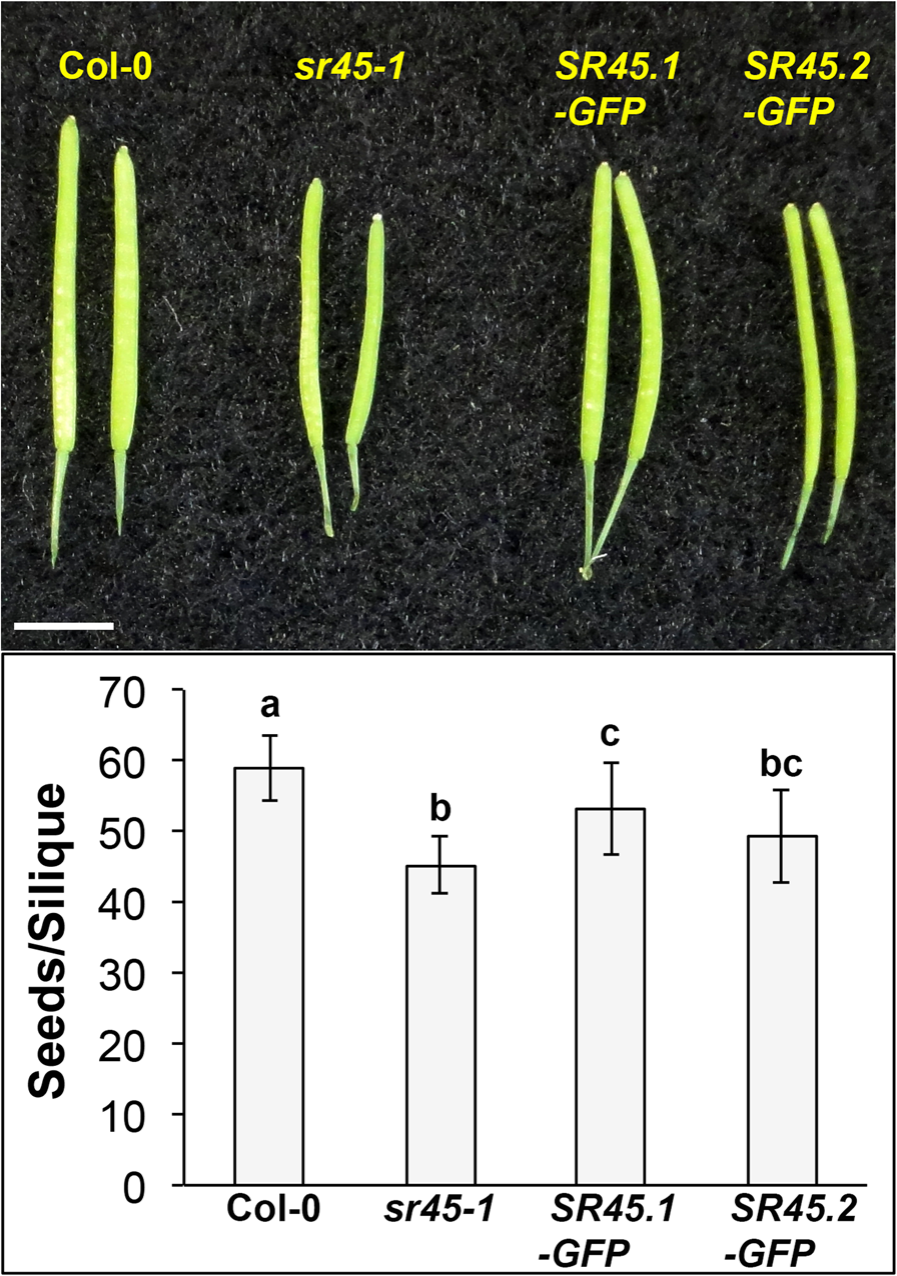
SR45 promotes seed yield. Top: A representative snapshot of siliques from Col-0, *sr45–1*, *SR45.1-GFP/sr45–1* and *SR45.2-GFP/sr45–1* plants. Scale bar = 5 mm. Bottom: A comparison of seed number per silique among Col-0, *sr45–1*, *SR45.1-GFP/sr45–1* and *SR45.2-GFP/sr45–1* lines*. n* = 17. Error bars present standard deviations. Letters a-c denote statistically significant difference analyzed by One-way ANOVA followed by Tukey’s t-test. *p* < 0.05

During flower development, petals protect plant reproductive organs and contribute to pollination success by attracting pollinators in the wild. The *sr45–1* mutant exhibits a significantly narrower flower petal compared to the wild type [2, 3]. This phenotype was mostly recovered by SR45.1-GFP, but not SR45.2-GFP [3]. After fertilization, the *sr45–1* mutant had a reduction in seed yield (an average of 45 vs. 59 seeds per silique, 76% of Col-0), and the mutant silique was visibly shorter. This defect in the *sr45–1* mutant was significantly improved by SR45.1-GFP, to 90% of Col-0, but not SR45.2-GFP (83% of Col-0) (Fig. 1). These observations led to the hypothesis that SR45 promotes gene function in flower and seed development. To test this hypothesis, we first performed RNA-seq with three biological replicates of inflorescence from Col-0 and *sr45–1* (Additional file 1: Figure S1)*.*

**Fig. 2:**
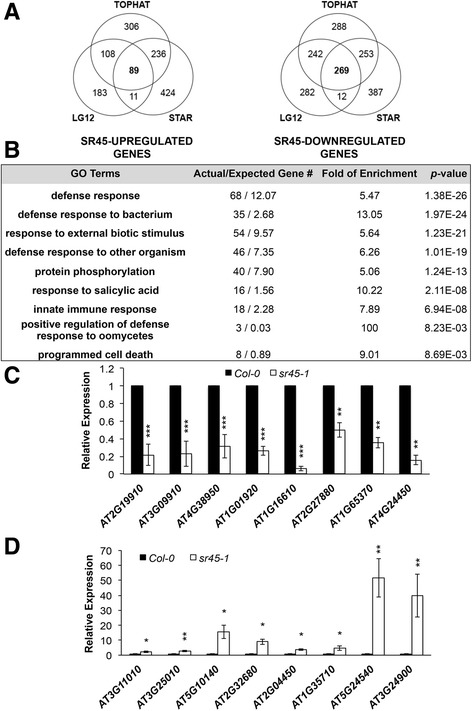
Defense genes are highly enriched in SR45-downregulated genes. **a**. Venn Diagrams showing SR45-upregulated and -downregulated genes identified by three different pipelines. **b**. A sample of GO enrichment analysis for SR45-downregulated genes that are commonly identified by all three pipelines. Fold of enrichment = ratio of Actual/Expected Gene numbers. **c**-**d**. qRT-PCR confirmation of selected SR45-differentially regulated genes. Relative expression of each gene is defined as 1 in Col-0. *GAPDH* was used as control. Poly(A)-mRNAs prepared from inflorescence were used to perform qRT-PCR in each sample as described in Methods. *n =* 3. Error bars present standard deviations. Student t-test was used to compare the relative expression between Col-0 and *sr45–1.* * *p <* 0.05, ** *p <* 0.01, *** *p <* 0.001

High quality Illumina Hiseq 50-nucleotide reads were aligned to the Arabidopsis genome (Tair 10). We used three different pipelines (Tophat2 [17], Splicing Transcripts Alignment Reference (STAR) [18] and Lasergene version 12 (LG12)) in order to eliminate pipeline-dependent bias during read alignment (Additional file 2: Figure S2). A quality control step coupled with trimming region with biased base composition ensured that only high-quality reads were used for subsequent analysis (Additional file 2: Figure S2). Uniquely mapped reads were assigned to a genome locus based on the highest probability. Multimapping reads were assigned using criteria specific to each pipeline (see Methods). Reads with no match were not used for locus assignment. The three pipelines yielded similar numbers of total mapped reads (Additional file 3: Table S1). The correlations among all resulting mappings were examined by pair-wise comparisons. The resulting R^2^ values showed a high correlation among libraries from the same genetic background (Additional file 4: Figure S3). The assembly files were then filtered by same criteria, fold change ≥2 and false discovery rate (*FDR*) < 0.1, to yield SR45 differentially regulated (SDR) genes. A total of 739, 760 and 391 SR45-upregulated genes, and a total of 1052, 921 and 805 SR45-downregulated genes were identified by Tophat2, STAR and LG12, respectively (Additional file 5: Table S2). After comparing the identity of these genes from all three pipelines, a total of 89 common genes were determined to be upregulated by SR45, and a total of 269 common genes were determined to be downregulated by SR45 (Fig. 2a). While many positive gene candidates were no doubt excluded by applying such a highly stringent filter, combining the three different pipelines reduced the chance of false positives due to pipeline-specific bias during the assembly process, and provides us with a robust set of common genes that are up-regulated or down-regulated by SR45 with high confidence. Therefore, we followed up with these common genes in the subsequent analysis.

No significant enrichment of gene ontology (GO) categories was observed for SR45-upregulated genes (those with reduced expression in the *sr45–1* mutant), which suggests that SR45 promotes the expression of genes in diverse pathways. However, several GO categories were significantly enriched with high confidence among SR45-downregulated genes (those with elevated expression in the *sr45–1* mutant) (Fig. 2b). When we compared the GO enrichment pattern in all analyses (Tophat2, STAR, LG12) individually (Additional file 6: Table S3), we found that all of the GO terms enriched in the common gene set showed similar or much better enrichment than was seen in the three individual pipelines. This suggests that the common gene set does indeed contain reduced background noise. However, the *p-*values for each GO category in the common gene set were slightly higher in most categories than in the individual pipelines due to a smaller number of the remaining genes in the common gene set.

The 89 SR45-upregulated genes are involved in diverse biological processes, from metabolism (*Glucose-6-phosphate 1-dehydrogenase 4, isocitrate lyase,* and *GDSL esterase*), transport (*peptide transporter 4, sodium/metabolite cotransporter BASS5* and *BASS6*), auxin biosynthesis (*YUC4*), kinases (*MAPKKK21* and *GWD2*) to transcriptional/post-transcriptional regulation (*RDR3*, *SR45, CID9*) (Additional file 5: Table S2). Selected genes were confirmed by qPCR (Fig. 2c). Despite the floral phenotype of *sr45–1*, none of these genes are known to directly or specifically contribute to flower or seed development. However, we think it is likely that most, if not all, of these SR45-upregulated genes are not primary targets of SR45. Since SR45 has been recognized as a splicing activator, it is plausible that SR45-dependent alternative splicing more directly acts on genes that in turn regulate flower and seed development.

Unexpectedly, we find that defense response genes are highly enriched among SR45-downregulated genes (68 of the 269). This number represents a 5.47 fold enrichment with the highest confidence (*p-*value = 1.38E-26) among all GO categories (Fig. 2b, Additional file 6: Table S3). The greatest enrichments with high confidence are found for subcategories *defense response to bacterium* (13.05, *p-*value = 1.97E-24) and *response to salicylic acid* (10.22, *p-*value = 2.11E-08) (Fig. 2b, Additional file 6: Table S3). Known defense marker genes (*PR1* and *PR5*), salicylic acid (SA) pathway genes (*ACD6* and *PAD4*), and LRR-receptor like protein genes (*RLP34* and *RLP41*) were all confirmed to have statistically significantly higher expression in the *sr45–1* mutant than the Col-0 wild type (Figs. 2d and 6k)*.* This suggests that SR45 plays a role in repressing the defense network when plants are not facing pathogen challenges. In addition, genes involved in protein phosphorylation, especially kinases, are also significantly overrepresented in the SR45-downregulated genes with a fold of enrichment of 5.06 and a *p-*value of 1.24E-13. These kinase genes include those coding for 11 proteins with leucine-rich repeat (LRR) and 20 cysteine-rich receptor-like protein kinases (CRKs) (Additional file 5: Table S2). To-date, a total of 44 CRKs have been identified in Arabidopsis and some are induced by ROS signaling, pathogen and SA [19, 20]. Twenty of them (about half) are induced in the *sr45–1* mutant, suggesting that SR45 may suppress a mechanism(s) that can trigger the simultaneous expression of many CRK genes during flower and/or seed development. In all 68 defense genes annotated by PANTHER, at least one W-box was found statistically significantly more highly presented in the −500 bps upstream genomic region compared to the genome background (TTTGAC with a *p-*value of 1.47E-80, TTGACC with a *p-*value of 0.00E + 00, and/or TTGACT with a *p-*value of 0.00E + 00) (Additional file 5: Table S2). This strongly suggests that the expression of this subset of defense genes may be controlled by a small set of WRKY transcription factors, and these WRKY transcription factors bind to sequences in the target genes better in the *sr45–1* mutant. The large number of plant defense genes present in the SR45-downregulated gene group led us to the hypothesis that SR45 negatively regulate innate immunity in Arabidopsis.

### SR45-dependent alternative splicing events in inflorescence

**Fig. 3:**
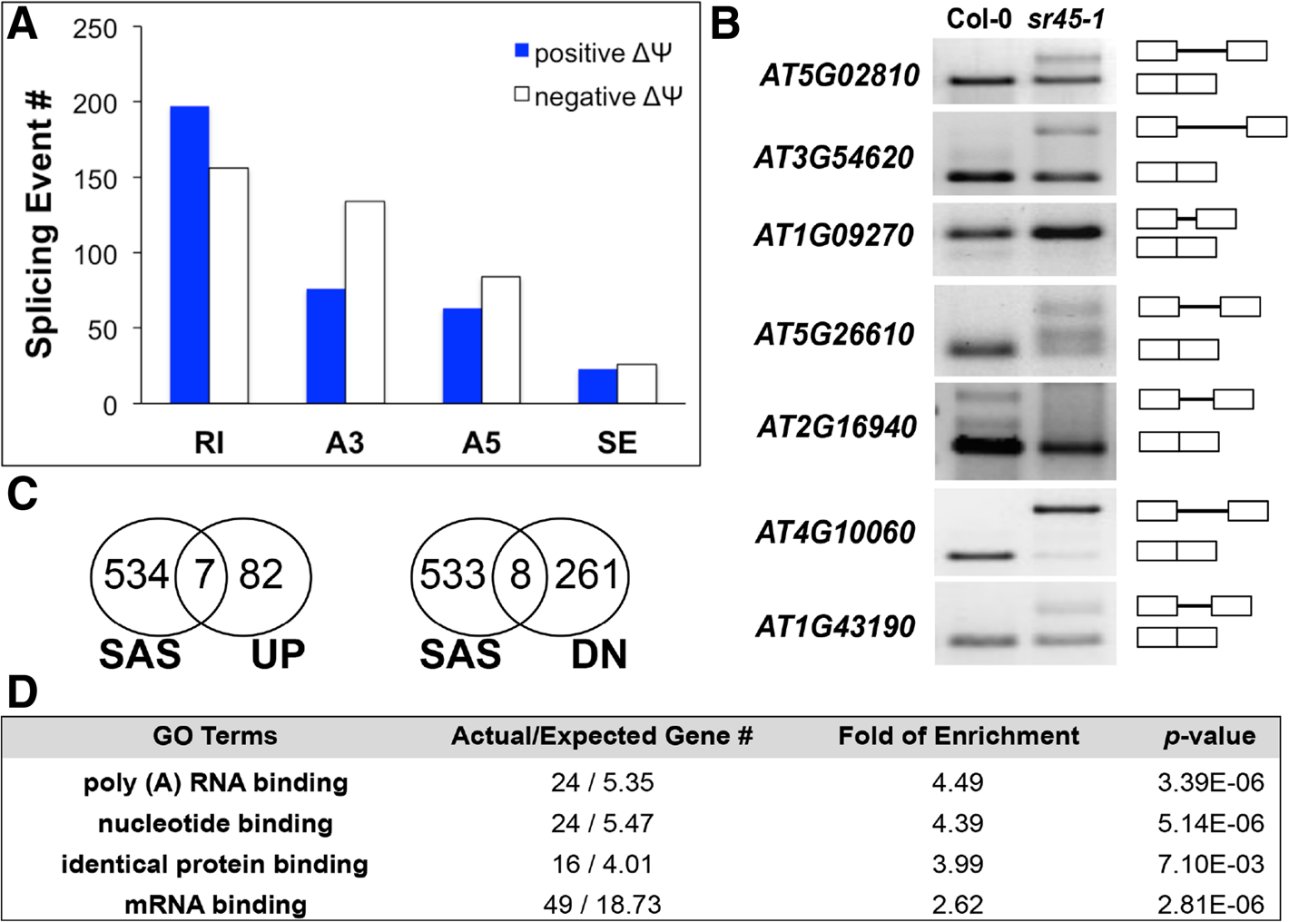
SR45-dependent alternative splicing (SAS) events. **a**. A comparison of different splicing events. *Ψ*: Percentage of Splice In. Δ*Ψ* = *Ψ*_*sr45–1*_ – *Ψ*_Col-0_. **b**. RT-PCR confirmation of selected alternative splicing events. **c**. Venn Diagrams showing SR45-dependent alternatively spliced genes that also upregulated or downregulated by SR45. **d**. GO enrichment analysis for SR45-dependent alternatively spliced (SAS) genes with a fold of enrichment greater than 2

SR45 regulates pre-mRNA splicing of target transcripts [2, 7, 21, 22]. Discovering SR45-dependent alternative splicing (SAS) events can shed light on how SR45 regulates genes that function in flower and seed development. We quantified isoforms annotated in the AtRTD v2_QUASI (AtRTD2) transcriptome [23] by using *Salmon* [24]. We obtained information on confirmed alternatively spliced isoforms and quantified the transcript abundance. Isoform abundance values were used by SUPPA2.0.0 [25] to estimate *Percentage Spliced-In* (*ψ*) values for each alternative splicing event. We then filtered the dataset with a combined criteria of *p-*value <0.05, and total mean TPM ≥ 10 (Additional file 7: Figure S4). A total of 759 splicing events met these criteria. Among these 759 events, 359 have a higher *ψ* value in *sr45–1* than Col-0 (Δ*ψ* > 0). The remaining 400 events have a higher *ψ* value in Col-0 than *sr45–1* (Δ*ψ* < 0). A further breakdown revealed that most SAS events are retained intron (RI), followed by alternative 3′ splice site (A3) and 5′ splice site (A5), with skipped exon (SE) being the least. There are more RI events associated with positive Δ*ψ* (meaning that SR45 mediates splicing in the wild-type), while more A3 and A5 events associated with a negative Δ*ψ*. The number of events for positive and negative Δ*ψ* is comparable for SE (Fig. 3A, Additional file 8: Table S4). We selected nine SAS events to validate. Seven of them were confirmed by rtPCR (Fig. 3b).

These 759 SAS events are located in 542 genes within which only 7 (SAS_UP) are upregulated by SR45 and 8 (SAS_DN) are downregulated by SR45 (Fig. 3C). Among the 8 SAS_DN genes, two genes (*WRKY18*, and *GRP3*) are responsive to pathogen or SA. Most SAS events do not result in overall changes in gene expression at the level of total RNA abundance. Among these 542 SAS genes, transcripts encoding mRNA-binding proteins are significantly enriched (Fig. 3D). The function of these genes includes splicing regulation (*RS41, RSZ22* and *SCL30A*), regulation of polyadenylation (*PAB8* and RBP47C), stress granule formation (*UBP1B*), RNA degradation (*DCP2*), translation (*RPS1, RPL13AB, RPL13B* and *RPL15B*), and defense response (*UBA2C*). This indicates that SR45 can regulate diverse aspects of mRNA metabolism through its effects on alternative splicing. It is not known whether SR45 acts directly or indirectly on the splicing of these genes. Although DCP2 has been indicated in post-embryonic development [26], GO enrichment analysis does not support a prominent connection to flower and seed development. Possibilities still remain that SR45 regulates the viability and/or splicing of associated transcripts, then the product of these associated transcripts regulate their targets post transcriptionally.

### SR45 associates with a subset of reproduction-regulating transcripts in inflorescence

**Fig. 4:**
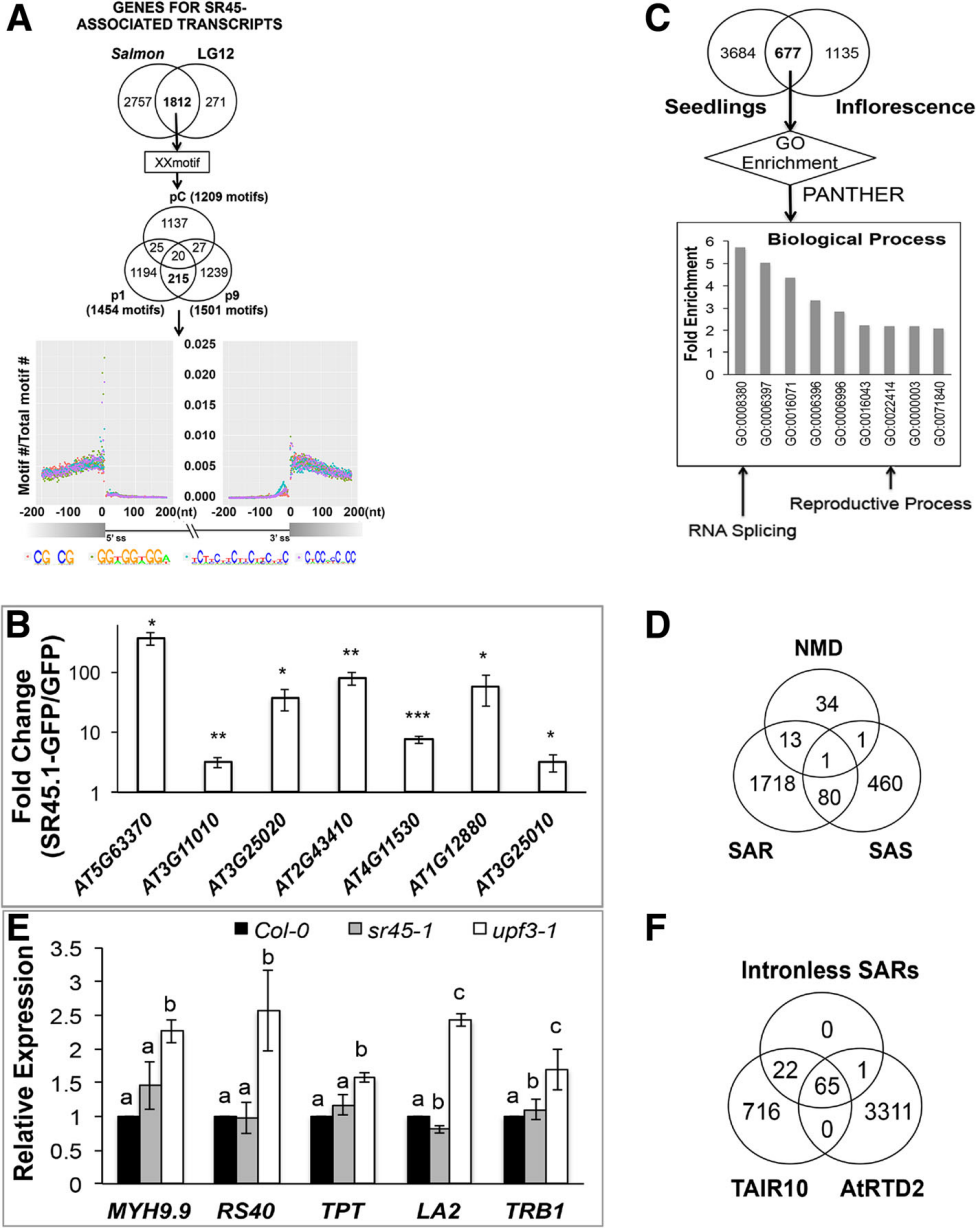
Discovery of SR45-associated transcripts and motifs in inflorescence. **a** Discovery of enriched motifs with highest occurrence in SR45-associated transcripts. SAR genes identified by Salmon and LG12 separately were compared, and the common genes were submitted to XXmotif to solicit enriched motifs in control (pC) and two independent SR45.1-GFP lines (p1 & p9). The 215 common motifs between p1 and p9, but not in pC were isolated and used for motif distribution analysis in the SAR genes. The locations of the top four highly occurring motifs around a splicing event were visualized as the ratio of the number of one particular Motif at a particular location /the number of total motifs observed in this particular location. **b** qPCR confirmation of SARs. *n* = 3. Error bars present Standard Error of Mean (SEM). * *p* < 0.05; ** *p* < 0.01; *** *p* < 0.001. **c** Venn diagram showing a comparison of SAR genes between seedling (Xing, 2015) and inflorescence and their GO enrichment. All selected GO terms have *p-value* < 1.0E-05 and fold of enrichment >2.0. **d** Venn Diagram showing SAR genes that are also subject to SR45-dependent alternative splicing, and/or NMD. **e** qPCR confirmation of SAR examples of NMD targets. *GAPDH* was used as control. *n* = 3. Letters a-c denote statistically significant difference among Col-0, *sr45–1* and *upf3–1* analyzed by Student t-test with Bonferroni correction. *p* < 0.05. **f** Intronless SARs identified by TAIR10 and/or AtRTD2

In order to identify SR45-associated transcripts in inflorescence, RIP-seq analysis was conducted with a GFP control line [16] and two SR45.1-GFP transgenic lines that we established previously (OX1–1 and OX1–9) [3]. See Additional file 1: Figure S1. The RIP method was adopted from the co-IP protocol we used to obtain SR45-associated proteins in vivo [6] with a modification at the end to extract associated RNAs instead of proteins. High-quality reads were processed by two different pipelines, in which reads were either mapped to the genome (LG12), or directly used to quantify annotated transcripts (*Salmon*). See Additional file 9: Figure S6. All transcripts meeting the following criteria were selected by *Salmon* or LG12, separately: fold change ≥2, and *FDR* (for LG12) or *p-*value (for *Salmon*) < 0.1 in both transgenic lines compared to control. Salmon identified 4569 genes which transcripts meet the criteria, while LG12 returned 2083 qualified genes (Additional file 10: Table S5). A total of 1812 genes were shared by both pipelines (Fig. 4a). The transcripts from these genes were considered SR45-associated RNAs (SARs) in inflorescence. Selected SARs were confirmed by RIP-qPCR (Fig. 4b). Interestingly, more than one third (677 out of 1812, Fig. 4c) of these inflorescence SAR genes overlap with the SAR genes from seedlings [16]. Among these 677 common SAR genes, RNA splicing genes and reproductive process genes were significantly over-represented (*p-*value <1.0E-05) (Fig. 4c, Additional file 10: Table S5). The 69 genes with the annotation “reproductive process” encode proteins with diverse functions, including nucleic acid binding proteins, transporters, receptors, oxidoreductases, chaperons, calcium-binding proteins, hydrolases, and cytoskeletal proteins. Among all these 69 genes, nucleic acid binding proteins exhibited the highest percentage (31.90%, 22 out of 69, Additional file 10: Table S5). They bind to nucleic acid at different levels. A major group of them is involved in epigenetic regulation ranging from flowering, flower development to embryo development in seeds, such as *FPA, VERNALIZATION INDEPENDENT 4* (*VIP4*), *BLISTER* (*BL1*), *FORGETTER 1* (*FGT1*) (Additional file 10: Table S5). In addition, a total of 19 SAR genes are involved in RNA splicing. This includes transcripts encoding SR45 and three SR proteins, SR34, SR30 and SCL33; pre-mRNA processing factors (PRP39, PRP40A, PRP40B and PRP2), the U11/U12 subunit SNRNP65, cyclin-dependent kinase G1 (CDKG1) and an RNA helicase (RH42) (Additional file 10: Table S5). Interestingly, five of these 19 SARs (*U1-70k, CACTIN, SR34, SR30* and *SCL33*) encode SR45-associated proteins [7, 8, 11], suggesting cross-regulation by factors that act with SR45 to regulate other targets.

The next logical question is what the fates of these inflorescence SAR genes are. One possibility is alternative splicing, or they could be degraded via nonsense-mediated decay (NMD). We intersected the 1812 inflorescence SAR genes with 542 SAS genes and 49 known NMD targets [27] (Fig. 4d). A total of 14 inflorescence SAR genes are recognized as NMD targets, while a total of 81 inflorescence SAR genes have identified SR45-dependent alternative splicing events (Additional file 10: Table S5). This is a significant over-representation of NMD targets (*p* < 0.0001 by Fisher’s exact test), and we have confirmed that five of these genes show increased abundance in *upf3–1* plants which has impaired NMD (Fig. 4e), We expect the number of inflorescence SAR genes that are known to be NMD targets will increase as more research data becomes available. Nevertheless, this still leaves the majority of the inflorescence SARs unaffected by either alternative splicing or NMD. Indeed, of the 1812 inflorescence SAR genes, 66 (3.6%) of them have no introns according to the AtRTD2 annotation. This phenomenon has been observed in 7.8% of seedling SAR genes using TAIR10 annotation recently [16]. Although the two annotations differ with respect to intronless genes, 90% of genes annotated by TAIR10 as intronless are likewise described as intronless by AtRTD2, and the majority of intronless SARs found in this study are annotated as intronless in both TAIR10 and AtRTD2 (Fig. 4f). The discrepancy between the two experiments could reflect a real difference between inflorescence and seedling, some unconfirmed annotations in TAIR10, and/or the incompleteness of AtRTD2. However, both studies clearly indicate that SR45 binds to many transcripts that are neither spliced nor subjected to NMD.

### Over-representation of motifs related to GGNGG at sites of SR45 binding and regulation

In order to identify potential binding sites for SR45, we looked for motifs that are over-represented in SARs or near sites of SR45-dependent alternative splicing.

First, we submitted the plus (mRNA-like) strand of all reads from the 1812 SAR genes identified by both of our analysis pipelines (Fig. 4a) to XXmotif [28]. Significant motifs from this analysis (over-represented relative to expectation based on nucleotide frequencies) that were not also enriched in the control were related to GGNGGNGG, CCNCCNCC, CGNCG and CTYCTYCTYC (Fig. 4a and Additional file 11: Table S6). These motifs were all also highly over-represented in mRNAs relative to introns and intergenic regions. So we repeated this analysis, using the annotated Arabidopsis transcriptome as background (Additional file 12: Figure S7). This time, the most significant motifs were all purine-rich motifs related to GGNGG, GNGGA and GNGGNNG, suggesting that SR45 may directly bind to such motifs.

**Fig. 5:**
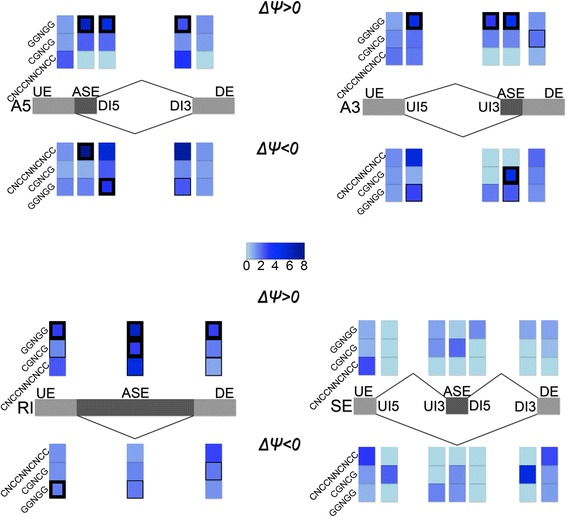
SR45-associated motif analysis. Heatmap showing relative enrichment in log2 scale of three SR45-associated motifs with positive *Δψ* (top) and negative *Δψ* (bottom). Relative enrichment is the ratio between enrichment in the alternatively spliced region of SARs to the corresponding regions within the entire transcriptome. Event classes: A5: alternative 5′ splice site, A3: alternative 3′ splice site, RI: retained intron, SE: skipped exon. Regions: UE: upstream exon, ASE: alternatively spliced exon, DE: downstream exon, UI5: upstream intron 5′ end, UI3: upstream intron 3′ end, DI5: downstream intron 5′ end, DI3: downstream intron 3′ end. Thin outlining denotes statistical significance of *p <* 0.01; thick outlining denotes statistical significance of *p <* 0.0001

To look for motifs enriched in the vicinity of alternative splicing sites whose usage is affected by *sr45–1*, motifs enriched in 50 nt. regions on both sides of splice sites involved in SAS events were identified. Over-representation was analyzed for each of the 20 regions shown in Fig. 5, using all alternative splicing events of the same class described in AtRTD2 as the control set. The most significant motifs from these 40 XXmotif analyses were examined manually and a set of similar motifs was selected for further analysis (see Methods). Then, the enrichment of specific occurrences of these similar motifs (the regular expressions GGNNNNGGNGG, GGNGGNGG, GGNGG, CNNCNNCNNCNNC, CNCCNNCNCC and CGNCGNCG) were examined for all AS events, and their enrichment vs. all alternative splicing events of the same type was calculated (Table 1, Fig. 5 and Additional file 11: Table S6). The greatest, and most significant, enrichments observed were for GGNGG and related motifs. Statistically significant enrichment of GGNGG was observed at 11 of the 20 regions examined, specifically the intron and exon sequences flanking alternative 5′ splice sites, alternative 3′ splice sites and retained introns (Table 1). Surprisingly, the motif was highly enriched for events with both positive and negative *Δψ*, and was enriched in both exons and introns flanking sites of SAS events. This suggests a strong correlation between these SAS events and the presence of SR45 at these motifs close to the SAS events.

**Table 1:** GGNGG enrichment at sites of SR45-dependent alternative splicing

		**All**	*ΔΨ* > 0			*ΔΨ* < 0		
Event Type	Site	GGNGG per kb.	GGNGG per kb.	Enrichment	*p*-value	GGNGG per kb.	Enrichment	*p*-value
A3	UI5	0.60	2.86	4.76	2.8E-20	1.70	2.84	3.0E-04
A3	UI3	0.96	2.91	3.02	1.3E-10	1.68	1.75	6.7E-02
A3	ASE	2.69	11.73	3.98	1.3E-16	6.48	2.32	2.7E-03
A5	ASE	2.49	15.33	6.49	1.4E-48	4.08	1.62	9.5E-02
A5	DI5	0.87	4.35	5.02	7.7E-26	2.87	3.32	1.2E-08
A5	DI3	0.99	2.52	2.55	2.3E-05	2.39	2.42	2.3E-04
RI	UE	2.30	6.74	2.94	8.1E-29	3.98	1.73	2.3E-05
RI	ASE	1.90	11.28	6.29	2.2E-62	2.43	1.85	7.3E-03
RI	DE	3.70	10.58	2.86	1.3E-42	4.81	1.30	2.9E-02

Enrichment statistics are shown only for GGNGG motif, and only for those sites that show significant enrichment (Additional file 11: Table S6) for either *ΔΨ* > 0, *ΔΨ* < 0, or both

### SR45 suppresses defense response upon pathogen infection

**Fig. 6:**
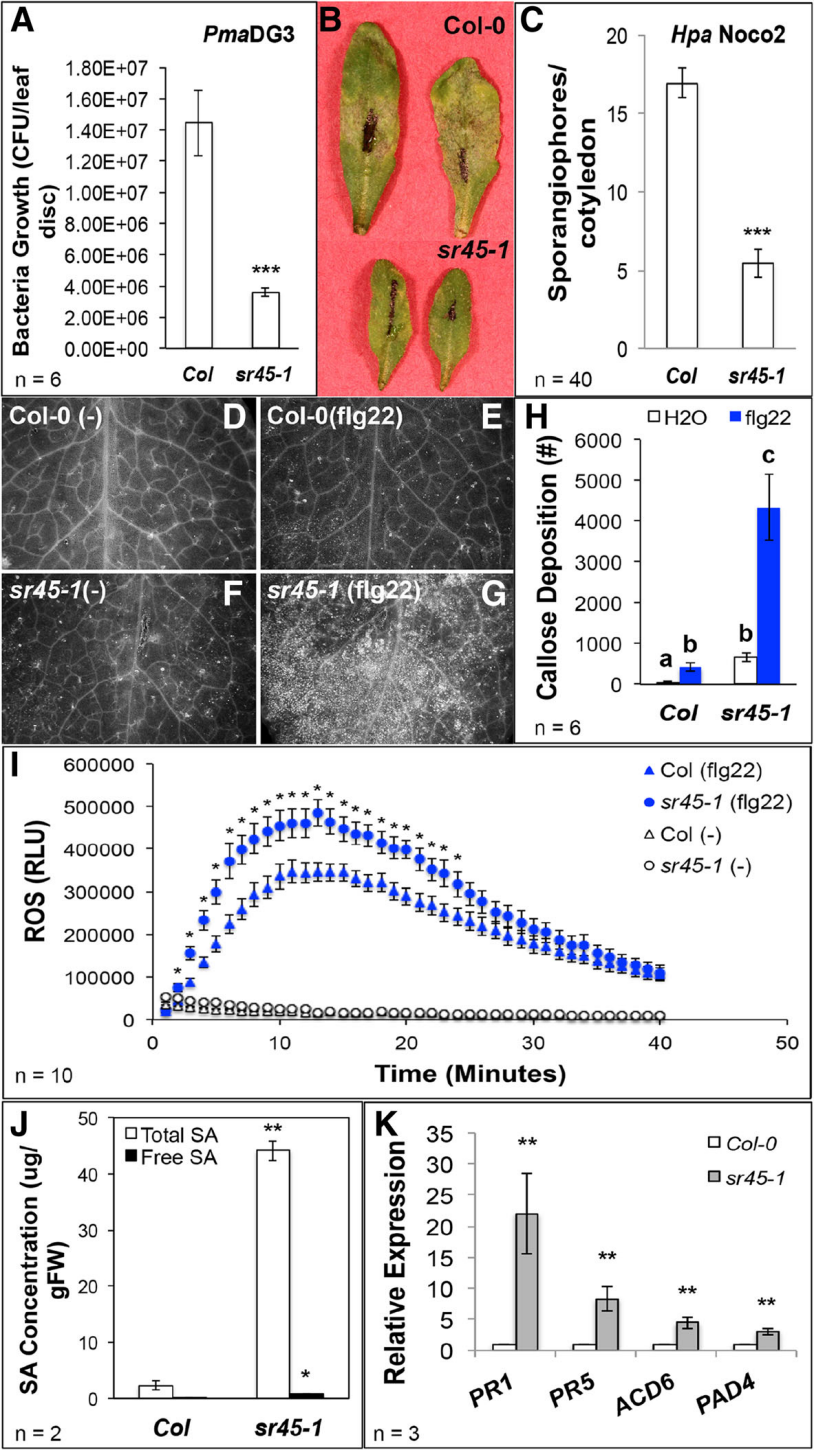
The *sr45–1* mutant exhibits elevated defense response. All error bars represent SEM. Student t-test was used for all statistical analyses with Bonferroni correction when comparing more than two groups. **a**-**b** *P. syringae Pma*DG3 inoculation test at 3 dpi. *n* = 6. ***: *p* < 0.001. **c** *H. parasitia* Noco2 infection test at 7dpi. *n* = 40. ***: *p* < 0.001. d-h: Callose deposition in response to 1 μM flg22. Callose deposition (**d**-**g**) is quantified by ImageJ (**h**). *n* = 6. Letters a-c denotes statistically significant difference (*p* < 0.05). **i** ROS production in response to 1 μM flg22. *n* = 10. *: *p* < 0.05. **j** Measurement of SA concentration. *n* = 2. *: *p* < 0.05; **: *p* < 0.01. K: qRT-PCR of SA pathway genes and defense marker genes in inflorescence tissue. *GAPDH* was used as control. *n* = 3. **: *p* < 0.01

To further investigate the defense role of SR45, we infected Col-0, *sr45–1, SR45.1-GFP* and *SR45.2-GFP* and* sr45-1* plants with different pathogens and found that the *sr45–1* mutant was more resistant to the bacterial pathogen *Pseudomonas syringae Pma*DG3 and the oomycete pathogen *Hyaloperonospora parasitica* Noco2 (Fig. 6a-c), both of which are virulent pathogen strains. In addition, the *sr45–1* mutant was more sensitive to flg22-induced host responses, including callose deposition (>10 fold increase) (Fig. 6d-h), and reactive oxygen species (ROS) production (a maximum of 60% increase) (Fig.6i). Moreover, both total SA and free SA are at much higher levels in the *sr45–1* mutant without any pathogen challenge (Fig. 6j). Both plant defense marker genes and SA signaling genes are expressed at a higher level in the *sr45–1* mutant compared to Col-0 (Fig. 6k). Together, these results strongly suggest that the *sr45–1* mutant has an elevated basal defense and supports our hypothesis that SR45 is a negative regulator of innate immunity in Arabidopsis.

## Discussion

Our analysis of SR45-dependent changes in transcript abundance has revealed that SR45 acts to reduce the expression of genes in defense against pathogens. Independent methods (immunoprecipitation of SR45-bound RNAs and analysis of differential alternative splicing) have also identified the same motif (GGNGG) as a likely site of SR45 binding. Furthermore, we find that the patterns of alternative splicing observed are inconsistent with simple models in which SR45 either activates or represses splicing.

The splicing factor SR45 has diverse roles, including developmental regulation and abiotic stress response [2, 3, 14, 15, 21]. Until recently [7, 11, 16, 22], evidence in describing mechanisms of SR45 functions has been rather limited. Various in vitro and in vivo studies have painted a rather focused protein network that SR45 is associated with, mainly including spliceosome components and SR proteins [7, 8, 11]. Although SR45 is orthologous to RNPS1 by sequence and domain structures [3], experimental confirmation of conserved function has been lacking. Because RNPS1 is a peripheral protein of the EJC, direct binding or indirect association of SR45 with the Arabidopsis EJC may be transient and difficult to catch in vivo*.* RNPS1 is also a component of two alternative complexes [29], ASAP (Acinus, RNPS1 and SAP18) and PSAP (Pinin, RNPS1 and SAP18). Although both complexes are involved in splicing regulation, the outcomes (whether a splicing event is promoted or inhibited) can be quite different dependent on what other protein factors they interact with [30]. With the revealed protein network in mind, it is of importance to investigate the identity, fate and function of immediate transcript targets of SR45 and SR45-associated protein networks in order to understand how SR45 regulates splicing in supporting the success of a biological system.

Here, we have studied the gene network that SR45 regulates by examining reproductive tissue for changes in alternative splicing and mRNA levels, and by asking which RNAs SR45 protein associates with in this tissue. We find that SR45 is associated with transcripts involved in reproductive process and RNA splicing, an observation that is consistent with the straightforward hypothesis that SR45, a splicing factor, may influence plant reproduction by acting on transcripts involved in reproduction. Previous studies have shown that the *sr45–1* mutant plant flowers late, produces an abnormally high level of *FLOWER LOCUS C* (*FLC*), a MADS-box repressor of vegetative-to-reproductive phase transition [2], and fails to sustain a normal level of DNA methylation in a DRM2-specific manner [14]. In addition, the *sr45–1* mutant produces fewer seeds (Fig. 1). *FLC* is regulated by Polycomb group (PcG)-mediated histone modification via an epigenetic reader recognizing cold memory element within the *FLC* gene [31]. Our study confirmed its increased expression in the *sr45–1* mutant (Fig. 2d). Transcripts of a suite of histone modifiers were identified in SARs in our study (Additional file 10: Table S5), among which *VIP4* has been shown to directly regulate the expression of *FLC* [32]. Moreover, *FLC* is also regulated by DNA methylation mediated by two RNA-binding proteins, FPA and FCA [33]. With a nonsense mutation in the first RRM3-coding sequence, the *fca-8* mutant failed to properly methylate *FLC,* flowered late and exhibit mild flower defects [33]. *FPA* is also a confirmed NMD target [27]. We confirmed that SR45 is associated with the *FPA* transcript (Fig. 4d). It is possible that SR45 directly regulates *FPA* at posttranscriptional level to ensure there is enough FPA protein around to mediate regulated-methylation at the *FLC* locus and other loci contributing to reproduction. Another interesting SAR is *HUA ENHANCED 4* (*HEN4*), a transcript codes for a KH domain RNA-binding protein that promote proper processing of *AGAMOUS* [34]. Defects in *HEN4* genes caused stamen transforming into petal [34]. The *sr45–1* mutant has mild alteration in the number of stamen and petals, and narrower petal morphology [2, 3]. It is possible that SR45 regulates flower development via genes such as *HEN4.* However, *HEN4* is neither identified as an SDR gene nor an SAS gene in this study. If *HEN4* indeed plays a key role in SR45-regulated flower development, it would be done through an unknown mechanism yet to be addressed. However, among the 7 SR45-upregulated SAS (SAS_UP) genes, *AT1G20120, AT1G23570* and *AT1G23580* exhibit flower-specific expression pattern (Additional file 13: Figure S5). When SR45 is associated with reproductive SARs, the protein product of some SARs could somehow affect splicing of these flower-specific SAS_UP genes and cause an upregulation in their expression during flower development. Although the role of SR45 on reproduction is not well understood, our study provided evidence for possible mechanisms that SR45 employs to regulate reproduction.

By associating with multiple transcripts from genes encoding proteins involved in splicing, including those which encode SR45-associated proteins (Additional file 10: Table S5) SR45 has the potential to regulate a vast splicing network. Although it is not clear if these RNA splicing regulator proteins directly bind to their own transcripts, this seemingly self-regulatory pathway has been observed in the case of *FCA* [35]*.* In addition, transcripts of most SR proteins are alternatively spliced [36], and SR proteins can interact with other proteins via their RS domains in vitro [8]. It seems to be an efficient strategy for SR45 to target SR protein transcripts that can in turn modulate further targets to increase the transcriptome complexity rapidly. Besides, some SARs are targets of NMD or differentially regulated by SR45 (Additional file 10: Table S5). All the evidence described above suggests a diverse fate map for SARs. This makes the analysis of SARs more complicated. Besides, association with SR45 can lead to different outcomes in a case-dependent manner. It is even more puzzling to see that 66 SARs are intronless according to AtRTD2. Since SR45 acts in a protein network, it is possible that SR45 is associated with these transcripts via other protein partner(s), some of which may play a role in transcription-splicing coupling [37]. The versatility of SR45-associated protein functions is likely a key to the intronless SARs. None of these 66 intronless SARs have identified SAS events, and none are upregulated by SR45. Only two of the intronless SARs (*AT3G25010* and *AT3G25020*) are down-regulated by SR45, both of which are involved in defense response. In fact, most SARs neither have SAS events, show SR45-dependent differential expression, nor are NMD targets. The reason for their association with SR45 remains to be investigated.

In search for potential SR45-binding sites in SARs, we found that motifs related to GGNGG (including GGNNNNGGNGG, GGNGGNGG and GGNGGNGG) are significantly over-represented near sites of alternative splicing affected by the *sr45–1* mutation (Fig. 5). These motifs were especially over-represented in both introns and exons adjacent to alternative splice sites (particularly, A5, A3 and RI). Enrichment was highest (and *p* values lowest) in alternative splicing events with a positive *Δψ*, meaning that these sequences were included at a higher rate in the *sr45–1* mutant, but events with negative *Δψ* also show a significant enrichment. The classical model for SR protein activation of splicing through Exonic Splicing Enhancers (ESEs) suggests that the SR protein binding sites would be enriched in exons whose inclusion depends on those SR proteins [38]. Indeed, GGNGG is enriched 1.4 fold (*p*-value = 0.63) in SE exons whose inclusion is reduced in the *sr45–1* mutant, and depleted four-fold (*p*-value = 0.18) in SE exons whose inclusion is increased in the mutant (Additional file 11: Table S6). However, neither of these enrichments is statistically significant. In contrast, the most significant enrichment (over 6-fold) occurs in the region between the two alternative 5′ splice sites that show a positive *ΔΨ* (Table 1; *p*-value = 1.4E-48). This is the opposite of what would be expected if SR45 were an activator of ESEs. Furthermore, GGNGG motifs are consistently enriched in splicing events affected by SR45 knockout, in both introns and exons, and independent of whether the effect on *ΔΨ* is positive or negative. Thus, we propose that SR45 acts to define the regions for splicing and facilitate the effect of other regulators, whose effect on alternative splicing is responsible for the outcome. This hypothesis is consistent with both the relatively mild (viable) phenotypes of *sr45–1* mutant and the fact that it is a (peripheral) member of the exon junction complex, which associates with all spliced transcripts.

This finding is similar, but not identical, to one of the hypothesized *cis-*elements in SAR found in seedlings [16] which resembles a comparable splicing enhancer sequence bound by a splicing activator, TRA2 in human [39]. It is also worth noting that our GGNGG motif is found both around promoted splicing events and around suppressed splicing events. It suggests that SR45 may cause activation or suppression of a near-by splicing event. This difference between our finding and prior findings will need to be further tested with in vitro binding assays with functional SR45 proteins. Since SR45 has one low complexity domain resides on each end of the protein [3], producing a large quantity of pure functional SR45 protein in heterogeneous hosts remains a challenge.

Several SARs that exhibit SAS events (for example, *AT4G16990, AT3G46530* and *AT1G588070*) code for NBS-LRR proteins. NBS-LRR proteins are R proteins that can regulate defense signaling pathways once pathogen effectors are detected [40]. Although their gene targets are still unknown, it has been suggested that these NBS-LRRs may associate with WRKY transcription factors to induce the expression of defense genes [41]. All the highly enriched defense genes downregulated by SR45 have W-box sequences in their promoter region up to −500 bps (Additional file 5: Table S2). Our data does not provide evidence for which WRKY proteins may be responsible for the expression of these defense genes in the *sr45–1* mutant. Although 6 *WRKYs* are identified as SARs in inflorescence, they do not seem to be differentially alternatively spliced or differentially expressed in our experiment. If they were the players that associate with the NBS-LRRs to trigger defense gene expression, SR45 may be regulating these *WRKY* SARs in ways other than alternative splicing and differential expression to suppress their functions in plant defense. One possibility would be that associating with SR45 makes them unavailable to the translation machinery, and they become available in the *sr45–1* null mutant. But this will need to be tested at protein level in the future. In additional, three non-SAR *WRKYs, WRKY18, WRKY75* and *WRKY50*, have been shown to play roles in plant defense [42–44]. They all exhibit a higher level of expression in the *sr45–1* mutant (Additional file 6: Table S3). Although it is not clear how they were promoted in the *sr45–1* mutant and whether they can regulate their own expression, they may be important players in binding to W-box and inducing the expression of other defense genes seen in the *sr45–1* mutant. One question needs to be addressed is that the overrepresentation of defense genes in the *sr45–1* mutant was from inflorescence. Although it is not fully understood what roles defense response genes play in inflorescence, evidence has suggested that stress-responsive genes are induced in sepals, and biotic stimuli-responsive genes are enriched in mature flowers [45]. If SR45 is a true repressor for plant defense, then the *sr45–1* null mutant should exhibit a higher resistance to pathogens and/or a more dramatic response to pathogen-derived elicitors. Indeed, the *sr45–1* mutant showed elevated basal defense to flg22 (Fig. 6d-i) and a stronger resistance to virulent pathogens (Fig. 6a-c). The current understanding on plant innate immunity is mostly focused on SA biosynthesis, accumulation and SA-mediated signaling pathway [46]. The *sr45–1* mutant accumulates significantly higher level of free SA and total SA compared to Col-0 (Fig. 6j). This big difference suggests that SR45 may post a greater suppression on the SA biosynthesis and/or less of catabolism than Col-0. Future studies on how SR45 affect SA metabolism will shed light on this significant difference that we observed. A second question is whether SR45 suppresses plant defense via SA-dependent or SA-independent pathways. An evaluation of pathogen response in the context of SA-deficient mutants will help explore the mechanism(s) that SR45 uses to suppress plant innate immunity.

When using comparative bioinformatics approaches to look for SDR genes, we noticed that each pipeline (Tophat2, STAR and LG12) produced a different set of candidate genes, among which the common genes are less than 50%. This observation underscores an issue that each computer algorithm is set up differently. During computing, the number of mapped reads is similar from pipeline to pipeline, but slight differences in read assignment algorithm can create large discrepancies as we reported in our experiment (Fig. 2a). A more in-depth comparison and fine-tuning in different pipelines will increase the power of bioinformatics in revealing highly confident and meaningful information in biology. At present time, the risk of misplacement of multimapping reads is more prominent in short reads than long reads.

## Conclusion

In conclusion, our study provides valuable information suggesting possible ways that SR45 and its associated protein network may affect reproduction and defense against pathogens. Motifs related to GGNGG are enriched both in SARs and near different types of SAS events, suggesting that SR45 recognizes this motif directly to regulate splicing in a way that defies simple categorization as an activator or repressor of splicing. Future work on confirming each mechanistic connection proposed here will increase understanding of how SR45 works with other proteins to fine tune the alternative splicing network and regulate crucial processes such as reproduction and plant defense.

## Methods

### Plant growth condition

All *Arabidopsis* plants used in this study are in *Colombia* (Col-0) background. Mutant plants, *sr45–1* (SALK_004132), *upf3–1* (SALK_025175) and *pad4–1* (CS3806) were originally from *Arabidopsis Biological Resource Center* (ABRC). Primers used to examine T-DNA insertion in *sr45–1* are as described previously and listed in Additional file 14: Table S7 [3]. All plants, other than plant defense assays, were grown in soil with 16/8 h photoperiod at 100 μmol m^−2^ s^−1^. Peter’s fertilizer (Griffin Greenhouse & Nursery Supplies, 67–2030) was applied at the concentration of 3 g L^−1^ before sowing the seeds. All plants were grown at 22 °C. Plants used for defense assays were grown in soil with 12/12 h photoperiod.

### Total RNA isolation and rtPCR

RNeasy Mini Plus Kit (Qiagen) was used to isolate total RNAs per manufacture’s instruction. About five micrograms of total RNA from each sample was used for reverse transcription with SMARTScribe Reverse Transcriptase from Clontech. Oligo d(T)_16_ was used as the primer. The resulting first strand cDNA was used for both rtPCR with Master Pro ThermoCycler (Eppendorf) or real-time quantitative PCR (qPCR) on Chromo 4 ThermoCycler (Bio-Rad Inc.). SYBR Green Super Mix (Invitrogen) was used to prepare all reactions for real-time qPCR reactions. *GAPDH* was used for normalization purpose. The splicing of SR protein genes was examined using gene specific primers. All primers used in this study are listed in Additional file 14: Table S7.

### RNA Immunoprecipitation (RIP)

Three biological replicates from each of the two SR45.1-GFP transgenic lines, OX1–1 and OX1–9, [3] and a GFP control line [16] were prepared for this experiment. Inflorescence tissue was harvested from 6 to 7 weeks old plants, ground into fine powder in liquid N_2_ and stored in −80°C freezer. A total of 5 mL of plant powder per sample was collected and used for an immunoprecipitation procedure as described previously [7]. A total of 8 units of RNAOut was used in all aqueous solutions. After Dynabead (Invitrogen)-GFP antibody (ab290 from Abcam) was incubated with nuclear lysate for 1 h, and washed with 1× washing buffer (Invitrogen) for 5 times, protein-bound RNA was extracted with Trizol reagent and chloroform. The aqueous phase was then isolated, and RNA was precipitated with 2.5 volumes of absolute ethanol at −20°C for 30 min. The mixture was then centrifuged at 4°C for 20 min. The tiny pellet was gently washed with 80% cold ethanol, centrifuged at 4°C for 20 min again before ethanol was carefully removed, and the trace amount of RNA were left air dry. The RNA was dissolved in 10 μL of RNase-free water.

### Library preparation and Illumina sequencing

For RNA-seq samples, total RNAs from three biological replicates of Col-0 and *sr45–1* was extracted using RNeasy Mini Kit (Qiagen). An amount of 5 μg of total RNA per sample was used to purify poly(A) mRNA using Magnetic mRNA Isolation Kit (NEB) for sequencing library construction. For RIP-seq samples, all 10 μL of dissolved RNA was used to construct sequencing library. All sequencing libraries were produced according to the method described by A. Hunt [47]. Illumina HiSeq was performed for single end 50-bp sequencing using Illumina Hiseq2500 at Genomic and Bioinformatic Core facility at University of Buffalo.

### Bioinformatics analysis

#### Differentially expressed genes

The quality of demultiplexed .fastq files were examined by FastQC_0.10.1. Trimmomatic [48] was used to remove 13 nt. from reads (for TopHat, STAR and Salmon analyses). The trimmed high quality .fastq data were then processed by three different workflows (TopHat2, STAR, and Lasergene v12) using default settings in parallel as illustrated in the flowcharts in Additional file 2: Figure S2 with the exception of intron size (5000 nt in TopHats and STAR). Differentially expressed genes were identified by the TopHat2, STAR and Lasergene v12 workflows independently first, then compared using “gplot” and “VennDiagram” R packages and “intersect” function in R. The gene set that was common to all three workflows was isolated for GO enrichment analysis using the Protein ANalysis THrough Evolutionary Relationships (PANTHER) [49].

### Alternative splicing events

The quality of demultiplexed .fastq files were examined by FastQC_0.10.1. The trimmed high quality .fastq data were analysed by *Salmon* (0.7.0) to quantify transcript abundance and to calculate values with AtRTD2 transcriptome [23] as reference (k-mer = 21), and SUPPA2 to calculate event inclusion levels (PSIs as *ψ* values) and statistical significance (*p*-value) between Col-0 and *sr45–1* as illustrated in Additional file 7: Figure S4. The total mean TPM of each splice event was calculated by adding the mean TPM of all isoforms involved in that event. Splicing events with *p*-value less than 0.05 and total mean TPM equal or greater than 10 were selected as SAS events. Genes harbouring these SAS events were sent to PANTHER for GO enrichment analysis.

### SR45-associated transcripts

The quality of demultiplexed .fastq files were examined by FastQC_0.10.1. The trimmed high quality .fastq data were then processed by two different workflows (*Salmon* and Lasergene v12) in parallel as illustrated in Additional file 9: Figure S6. For *Salmon* workflow, duplicated reads in pC, p1 and p9 fastq files were removed by PRINSEQ (0.20.4). *Salmon* (0.7.0) was used as described above to yield isoform level TPM which was used to quantify gene-level TPM in Tximport [50]. A student t-test was performed to yield *p-*values for fold change of p1/pC and p9/pC independently. For Lasergene v12 workflow, we used default setting for RNA-seq analysis in ArrayStar to obtain RPKM, fold change (p1/pC and p9/pC) and *FDR*. Transcripts with fold change no less than 2 and *p-*value (or *FDR*) less than 0.1 for both p1/pC and p9/pC were selected as SR45-associated transcripts in these two workflows independently first, then compared using “gplot” and “VennDiagram” R packages and “intersect” function in R. The gene set that was common to both workflows was sent to PANTHER for GO enrichment analysis.

The read abundance for genes of interest was visualized by Integrative Genome Viewer (IGV) or GeneVison Pro (Lasergene) by aligning unique .fastq files of pC, p1 and p9 with TAIR10 genome reference and AtRTD2 annotation with the maximum intron length 5000 and segment length 14. The sorted .bam files from three replicates of the same sample were merged and used for visualization.

### Highly enriched SR45-associated motifs

RIP-seq reads were mapped to the AtRTD2 transcriptome using Bowtie2. Unique reads from the 1812 genes that were identified as common SR45-associated transcripts were then used to identify enriched motifs using XXmotif [28] with default settings. The common motifs shared by both transgenic lines but not in the GFP negative control were identified by merging common motifs found in both p1 and p9 motif dataframes followed by a “which(!)” command to exclude the motifs also found in pC motif dataframe. The resulting motifs with highest occurrence rate were selected for distribution analysis as shown in Fig. 4a. Motifs with length less than or equal to 9 nt. over-represented in RIP-seq reads (relative to the complete sequence of the 1812 mRNAs to which they map, discussed in the text) were derived using XXmotif.

Motifs enriched adjacent to alternative splicing events showing differential usage in *sr45–1* were identified using XXmotif in 20 sets of sequences, using all alternative splicing events described in AtRTD2 as background. The 20 regions were: 50 nt. upstream of the more upstream 5′ splice site of two alternative 5′ splice sites (A5 UE), the region between two alternative 5′ splice sites (A5 ASE), 50 nt. downstream of the more downstream of two alternative 5′ splice sites (A5 DI5), 50 nt. upstream of the 3′ splice site shared by alternative 5′ splice sites (A5 DI3), first 50 nt. of the exon downstream of an intron with alternative 5′ splice sites (A5 DE); 50 nt. upstream of the 5′ splice site shared by alternative 3′ splice sites (A3 UE), 50 nt. downstream of the 5′ splice site shared by alternative 3′ splice sites (A3 UI5), 50 nt. upstream of the more upstream of two alternative 3′ splice sites (A3 UI3), the region between two alternative 3′ splice sites (A3 ASE), first 50 nt. of the exon downstream of more downstream 3′ splice site of two alternative 3′ splice sites (A3 DE); 50 nt. upstream 5′ splice site of a retained intron (RI UE), the retained intron, 50 nt. downstream of a retained intron (RI DE); 50 nt. upstream of the 5′ splice site of an intron flanking a skipped exon (SE UE), the first 50 nt. of an intron containing a skipped exon (SE UI5), the 50 nt. upstream of an alternatively spliced exon (SE UI3), the alternatively skipped exon (SE ASE), the 50 nt. downstream of an alternatively spliced exon (SE DI5), the last 50 nt. of an intron containing a skipped exon (SE DI3), and first 50 nt. downstream of the 3′ splice site of an intron flanking a skipped exon (SE DE); (Fig. 5). Inspection of all of these revealed a set of common motifs whose abundance in the same regions was quantified as regular expressions (Fig. 5 and Additional file 11: Table S6); statistical significance of enrichment was determined using a chi-squared test. The distribution of GGNGG motifs plotted in Additional file 12: Figure S7 was determined for statistically significant events (*p-*value <0.05) and non-significant events (*p*-value >0.15) with mean TPM > 20.

### Transcription factor binding motif analysis in upstream gene sequences

AGIs of all 269 SR45-downregulated genes or 68 highly enriched SR45-downregulated defense genes were used as query to obtain statistically significantly overrepresented DNA motifs using Statistical Motif Analysis in Promoter or Upstream Gene Sequence tool available from TAIR (www.arabidopsis.org).

### Statistics

To determine differentially expressed genes, False Discovery Rate (*FDR*) was used when appropriate. Student t-test was used in evaluating the statistical significance between two groups. For comparison of more than two groups, either student t-test followed by Bonferroni correction or One-way ANOVA followed by Tukey t-test was performed. The correlation of each assembled sequencing files was evaluated by R^2^ value in a pair-wise fashion. The comparison result was submitted to an R script to create the heat map visualization.

### Pathogen infection assays

The bacterial growth assay was done according to an established protocol [51] with modifications. A fresh culture of *Pseudomomas syringae* pv. *maculicola* (*Pma*) DG3 (a *recA* derivative of ES4326) was grown in King’s Broth over night at 30°C. The overnight culture was diluted to OD_600_ = 0.0001 in 10 mM of MgSO4. The forth to sixth leaves of 25-day old plants grown under 12/12 light period were infected with the diluted bacterial solution for three days before 4-mm diameter leaf discs were punched, ground for bacterial count.

For oomycete treatment, the *Hyaloperonospora arabidopsidis* (*Hpa*) Noco2 virulent stain was prepared and sprayed with a spore suspension (1 × 10^4^ spores/mL in water). Sporangiophores on both sides of cotyledons were counted to determine the level of resistance as described previously [52]. Both bacterial and oomycetes infection tests were repeated at least twice.

### ROS assay

Leaves from 5-week old plants were used to make 4 mm discs. The leaf discs were equilibrated in sterilized water in a 96-well plate with one disc per well, covered with foil and left in growth chamber overnight. Then one half of the leaf discs was treated with 1 μM of flg22 (PhytoTechnologies Lab). Fluorescence dye L-012 (Wako) was added to each well at the final concentration of 0.3 mM. The luminescence was measured immediately using an illuminometer at 458 nm. Readings from 12 leaf discs per sample were used for statistical analysis. This was repeated twice.

### Callose deposition analysis

Leaves from 4-week old plants were infiltrated with either 1 μM flg22 or H_2_O overnight, then treated with alcoholic lactophenol, examined under fluorescent dissection microscope as described previously [53]. Image from 6 leaves were taken, and fluorescent particles were counted using Image J. This was repeated twice.

### Salicylic acid measurements

Rosette leaves from 28-day old plants were ground in liquid nitrogen, and salicylic acid was extracted and analysed by HPLC as described previously [54]. It was repeated at least twice.

### Large datasets

All RNA-seq and RIP-seq reads are stored as a BioProject (PRJNA382852) at NCBI Sequence Read Archive (SRA).

## Additional files

Additional file 1: Figure S1. An illustration of the experimental design. (TIFF 38 kb)

Additional file 2: Figure S2. Flowcharts illustrating all three (TOPHAT, STAR and LG12) pipelines used in RNA-seq data analysis. (TIFF 247 kb)

Additional file 3: Table S1. A summary of assembly results. (XLSX 35 kb)

Additional file 4: Figure S3. A heat map showing a pair-wise comparison of all 24 RNA-seq and RIP-seq libraries by ranking of R^2^ values. (TIFF 201 kb)

Additional file 5: Table S2. Summary lists of SR45 differentially regulated (SDR) genes identified by three different pipelines. (XLSX 891 kb)

Additional file 6: Table S3. GO enrichment comparison for SR45-downregulated genes among three different pipelines. (XLSX 22 kb)

Additional file 7: Figure S4. A flowchart showing the pipeline used to identify alternative splicing events based on AtRTD2. (TIFF 258 kb)

Additional file 8: Table S4. SR45-dependent alternative splicing (SAS) events. (XLSX 210 kb)

Additional file 9: Figure S6. Flowcharts illustrating the two (Salmon & LG12) pipelines used in RIP-seq data analysis. (TIFF 250 kb)

Additional file 10: Table S5. Summary lists of genes that SR45-associated RNAs (SARs) were identified by two different pipelines. (XLSX 1461 kb)

Additional file 11: Table S6. A summary list of SR45-assocated motifs enriched at sites of alternative splicing regulation. (XLSX 37 kb)

Additional file 12: Figure S7. Over-represented SR45-associated motifs (SAMs) at sites of alternative splicing regulation. (TIFF 1398 kb)

Additional file 13: Figure S5. An AtGE Development panel showing the expression pattern of 7 AS_UP genes generated by AtGenExpress Visualization Tool (AVT). (TIFF 420 kb)

Additional file 14: Table S7. A summary of all primers used in this study. (XLSX 47 kb)

## Abbreviations

FDR: False Discovery Rate
LG12: Lasergene version 12
PANTHER: Protein ANalysis THrough Evolutionary Relationships
SAR: SR45-associated RNA
SAS: SR45-dependent alternative splicing
SDR: SR45 differentially regulated
TPM: Transcripts Per Kilobase Million
