# Supplementary figures and images for: Transcriptome analyses reveal SR45 to be a neutral splicing regulator and a suppressor of innate immunity in Arabidopsis thaliana
Source: BMC Genomics. 2017 Oct 11;18:772. doi: 10.1186/s12864-017-4183-7 (PMC5637254; doi:10.1186/s12864-017-4183-7)

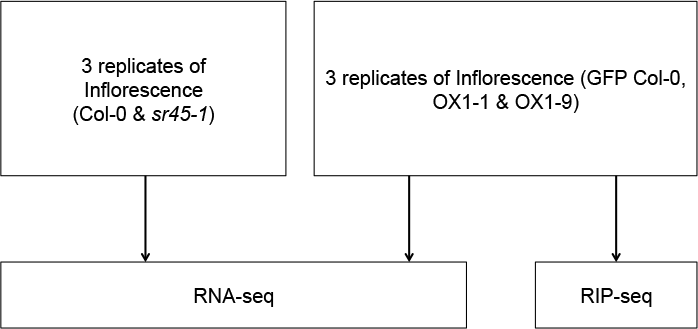

Supplement: Supplementary file 1 — An illustration of the experimental design. (TIFF 38 kb) [file 12864_2017_4183_MOESM1_ESM.tif]

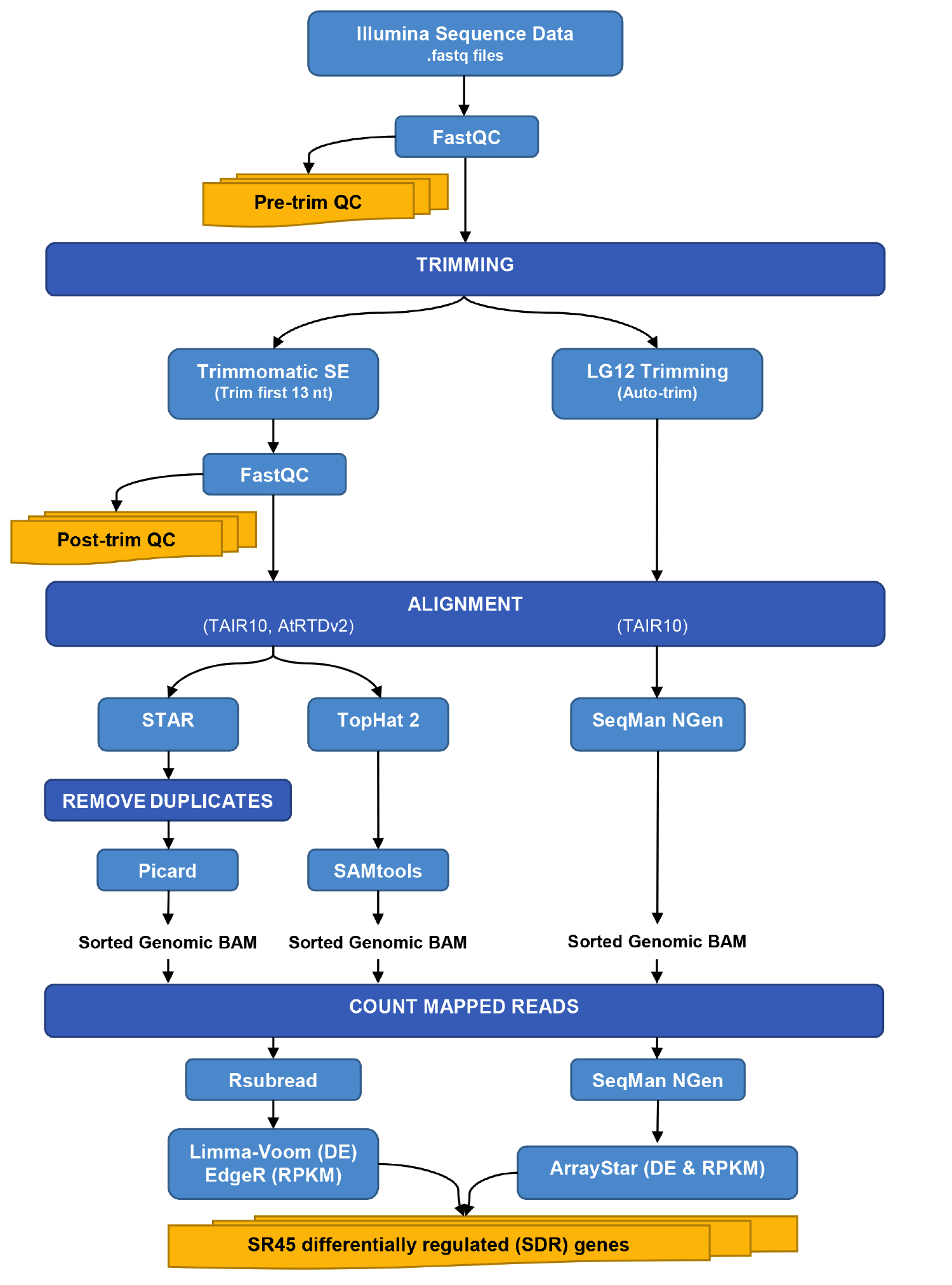

Supplement: Supplementary file 2 — Flowcharts illustrating all three (TOPHAT, STAR and LG12) pipelines used in RNA-seq data analysis. (TIFF 247 kb) [file 12864_2017_4183_MOESM2_ESM.tif]

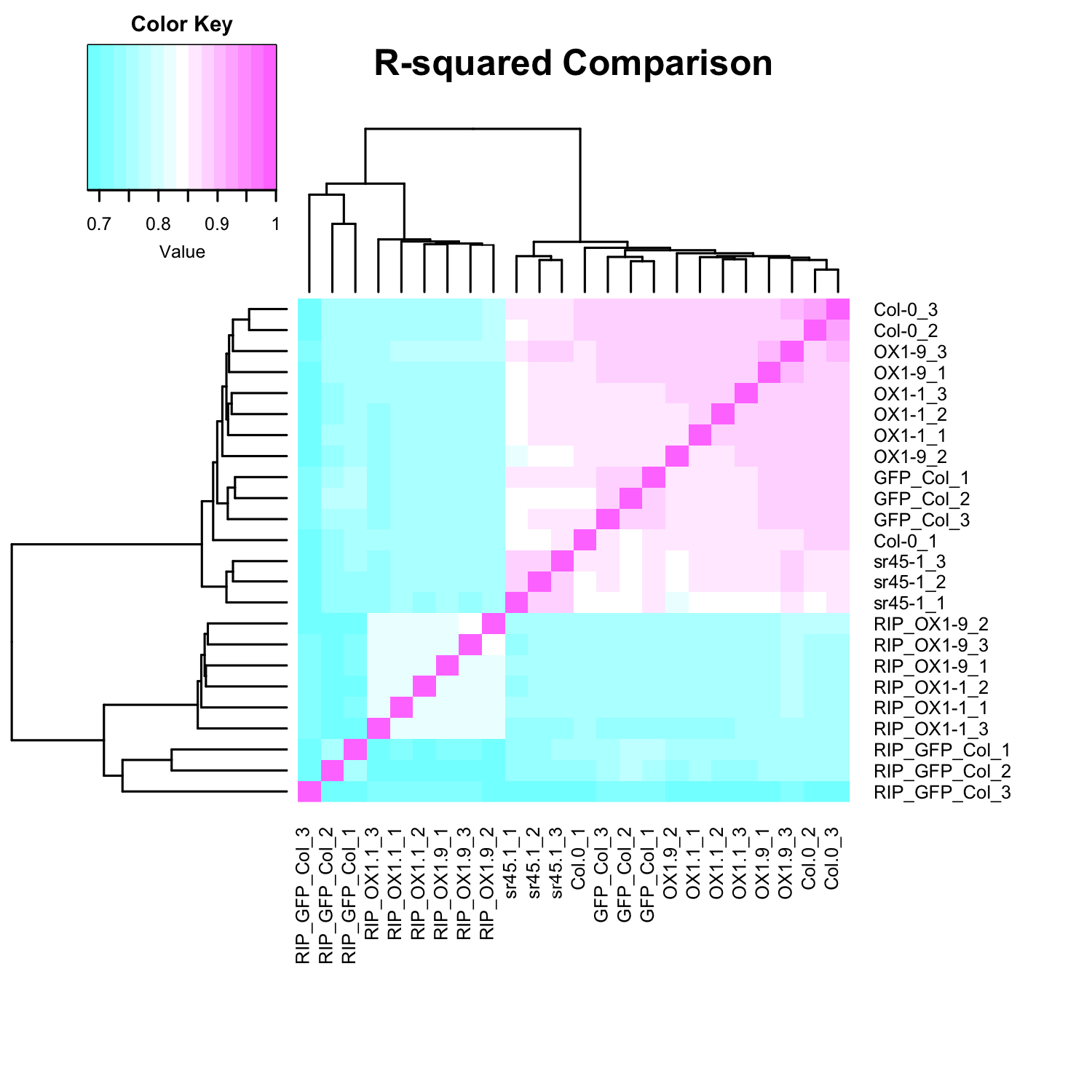

Supplement: Supplementary file 4 — A heat map showing a pair-wise comparison of all 24 RNA-seq and RIP-seq libraries by ranking of R2 values. (TIFF 201 kb) [file 12864_2017_4183_MOESM4_ESM.tif]

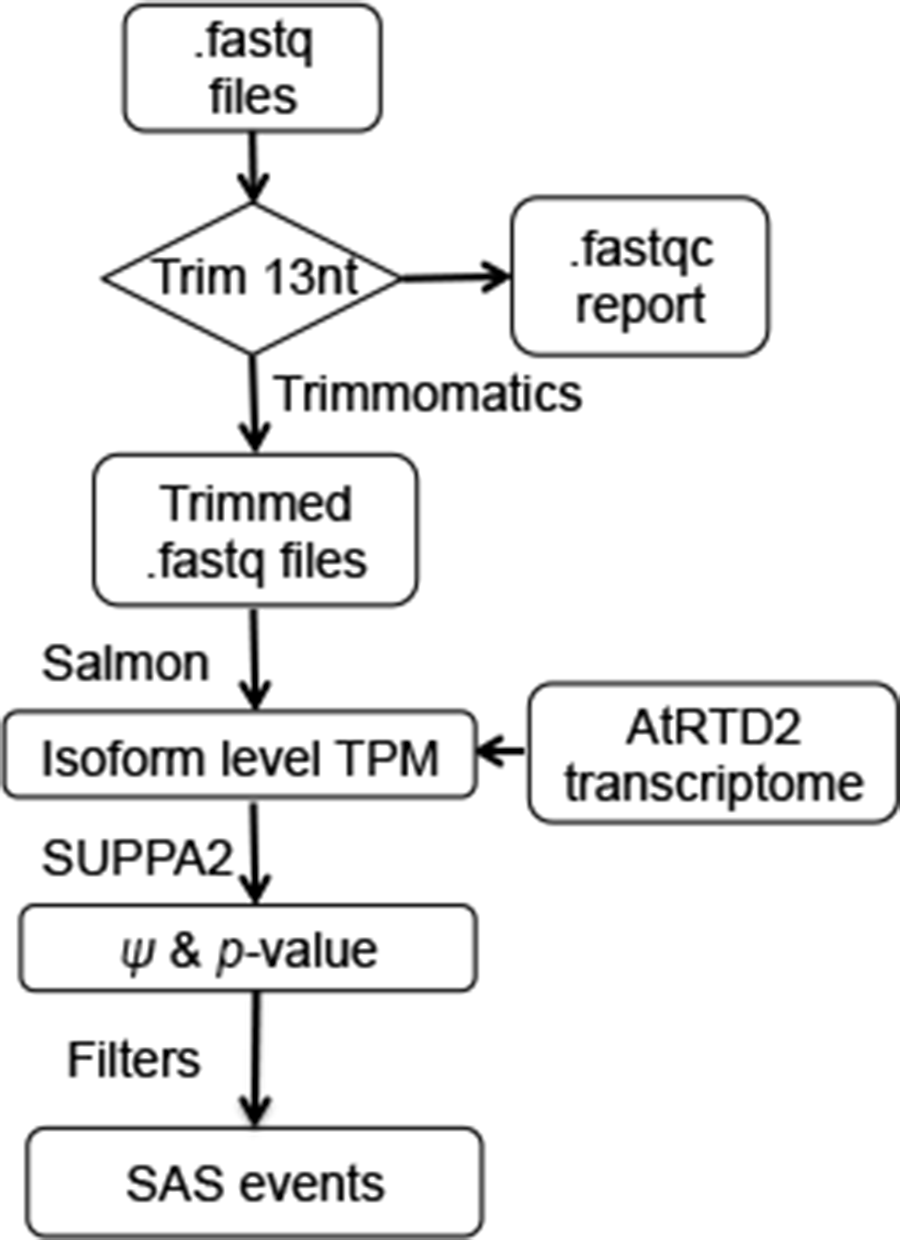

Supplement: Supplementary file 7 — A flowchart showing the pipeline used to identify alternative splicing events based on AtRTD2. (TIFF 258 kb) [file 12864_2017_4183_MOESM7_ESM.tif]

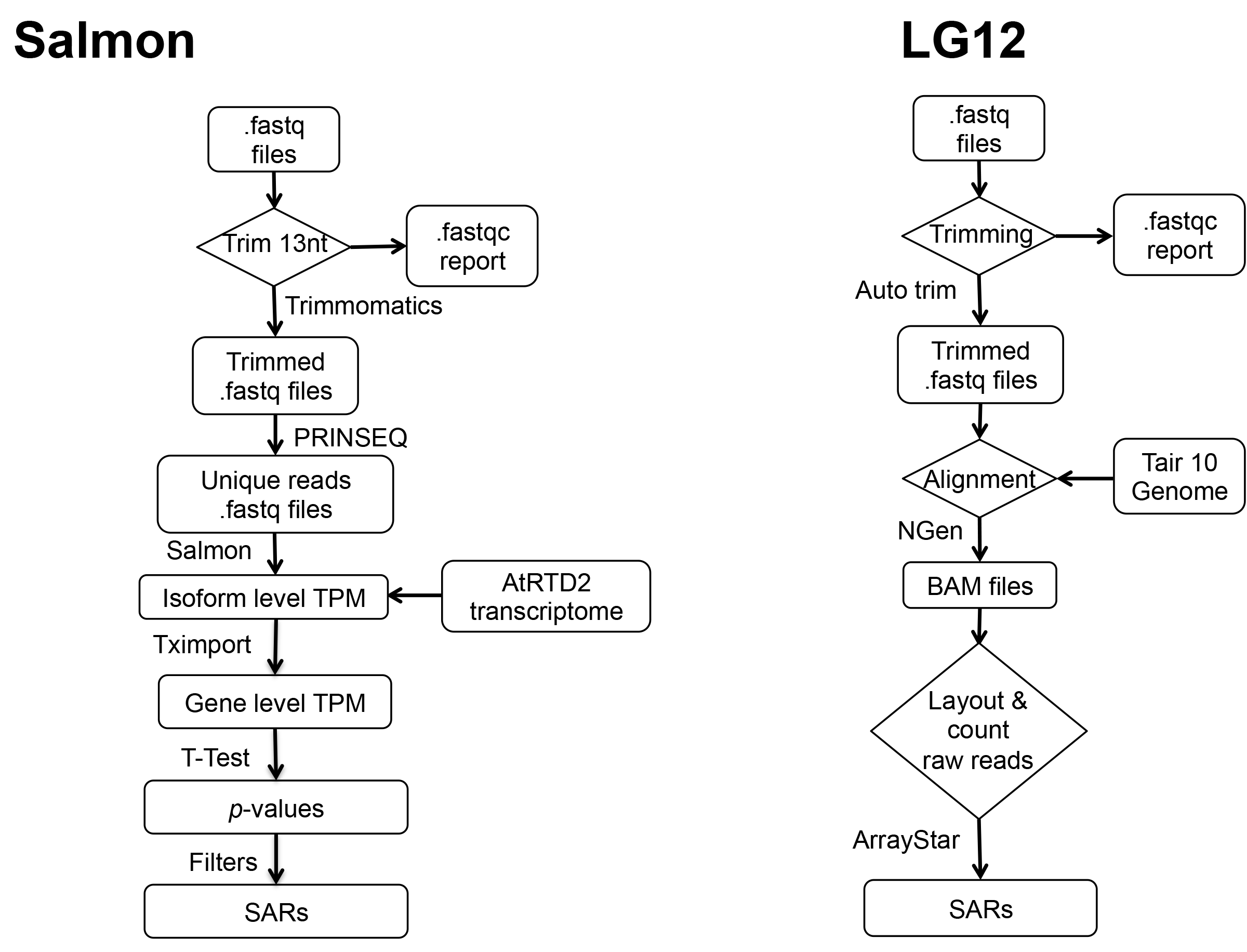

Supplement: Supplementary file 9 — Flowcharts illustrating the two (Salmon & LG12) pipelines used in RIP-seq data analysis. (TIFF 250 kb) [file 12864_2017_4183_MOESM9_ESM.tif]

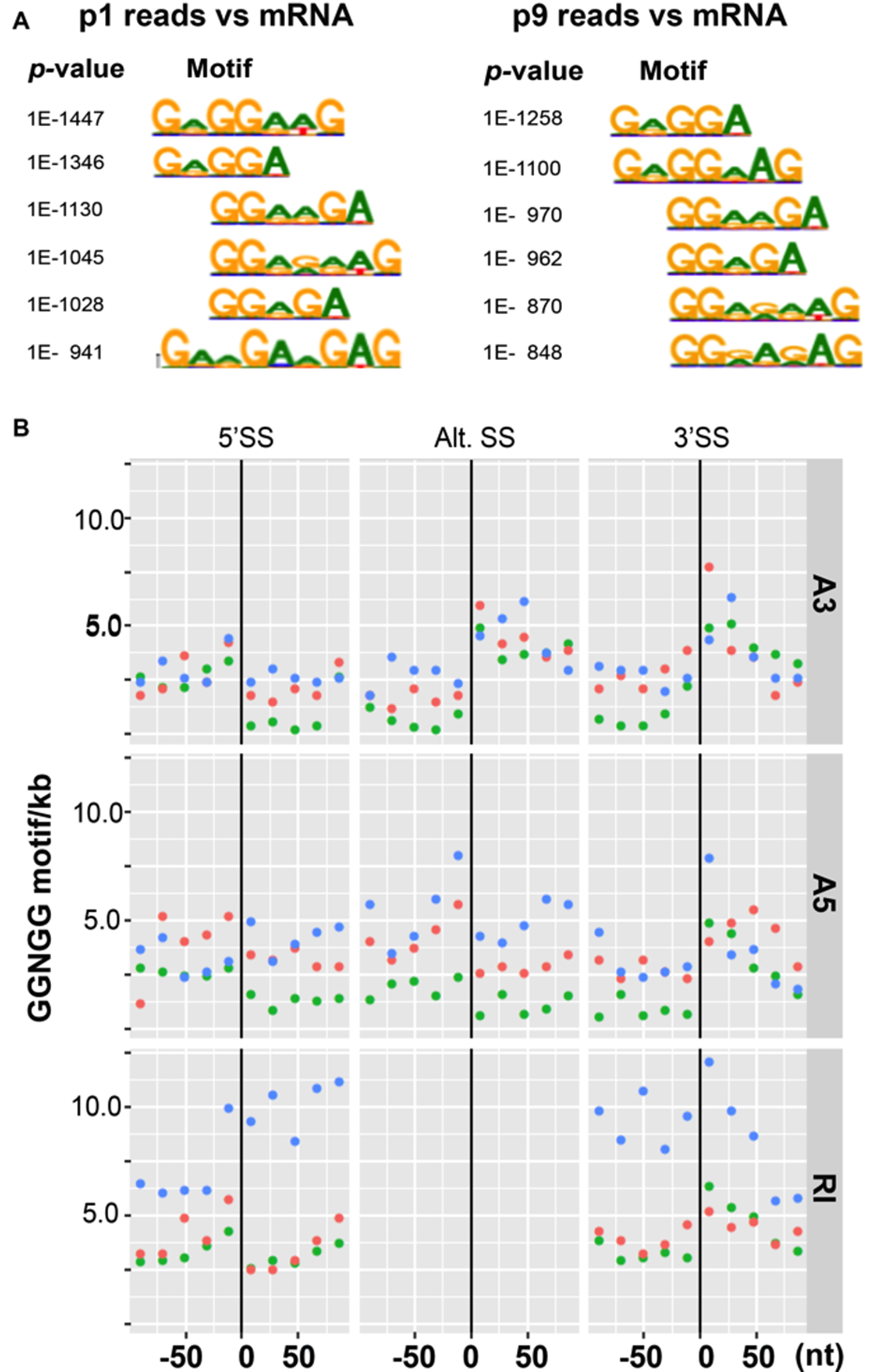

Supplement: Supplementary file 12 — Over-represented SR45-associated motifs (SAMs) at sites of alternative splicing regulation. (TIFF 1398 kb) [file 12864_2017_4183_MOESM12_ESM.tif]

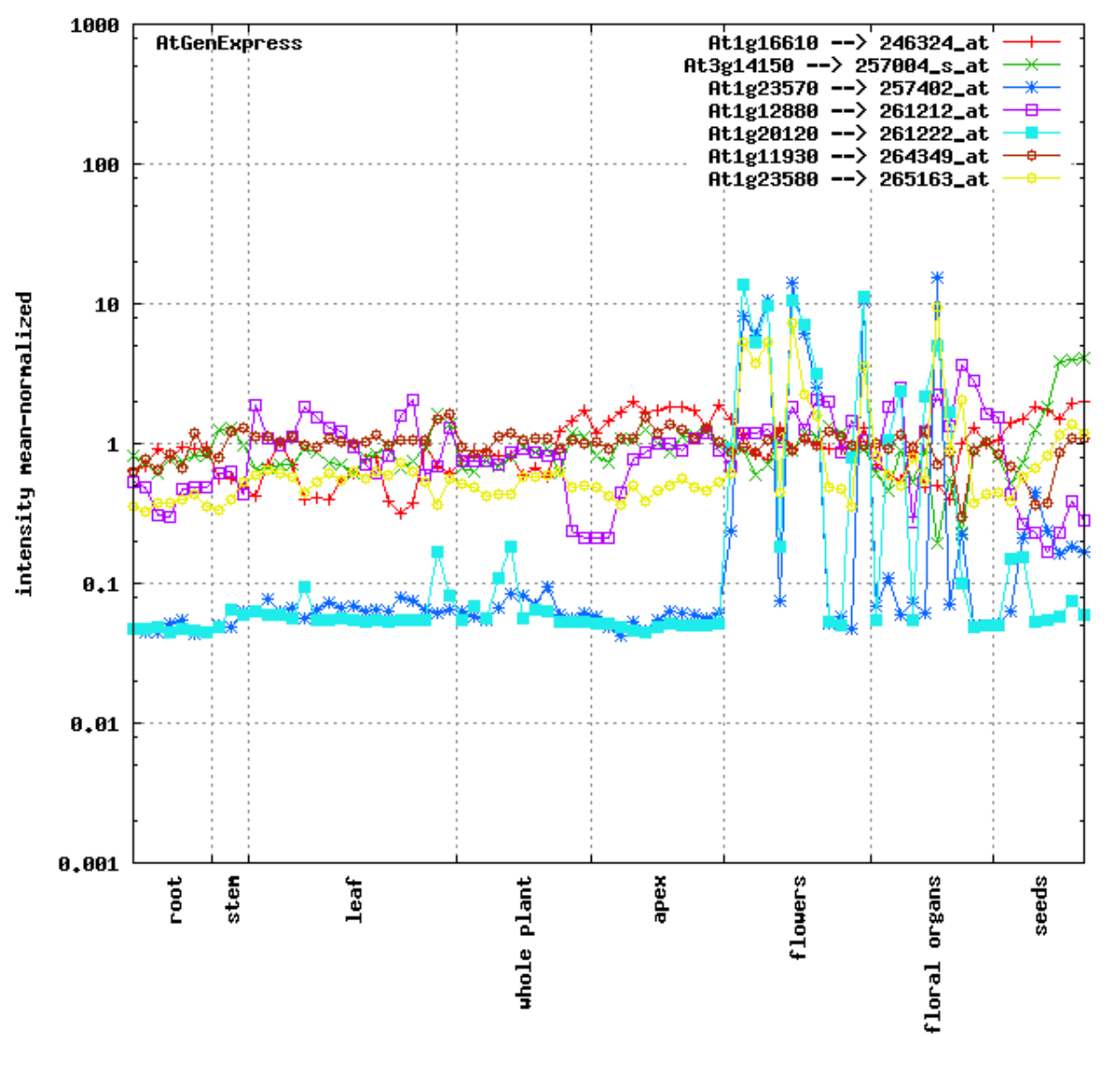

Supplement: Supplementary file 13 — An AtGE Development panel showing the expression pattern of 7 AS_UP genes generated by AtGenExpress Visualization Tool (AVT). (TIFF 420 kb) [file 12864_2017_4183_MOESM13_ESM.tif]
